# Noise in the intensive care unit and its influence on sleep quality: a multicenter observational study in Dutch intensive care units

**DOI:** 10.1186/s13054-018-2182-y

**Published:** 2018-10-05

**Authors:** Koen S. Simons, Eva Verweij, Paul M. C. Lemmens, Sam Jelfs, Munhum Park, Peter E. Spronk, Johannes P. C. Sonneveld, Hilde-Marieken Feijen, Marijke S. van der Steen, Armin G. Kohlrausch, Mark van den Boogaard, Cornelis P. C. de Jager

**Affiliations:** 1 Departement of Intensive Care, Jeroen Bosch Ziekenhuis, Henri Dunantstraat 1, 5223 GZ ‘s-Hertogenbosch, The Netherlands; 2 Philips Research, Eindhoven, The Netherlands; 3 Institute of Music, Science and Engineering, King Mongkut’s Institute of Technology Ladkrabang, Bangkok, Thailand; 4 Departement of Intensive Care, Gelre Ziekenhuizen, Apeldoorn, The Netherlands; 5 Departement of Intensive Care, Isala Klinieken, Zwolle, The Netherlands; 6 Departement of Intensive Care, Onze Lieve Vrouwe Gasthuis, Amsterdam, The Netherlands; 7 Departement of Intensive Care, Ziekenhuis Gelderse Vallei, Ede, The Netherlands; 8 Human-Technology Interaction Group, Technische Universiteit Eindhoven, Eindhoven, The Netherlands; 9 Departement of Intensive Care Research, Radboud University Medical Center, Nijmegen, The Netherlands

**Keywords:** Noise, Sleep quality, Intensive care unit, Critical illness, RCSQ

## Abstract

**Background:**

High noise levels in the intensive care unit (ICU) are a well-known problem. Little is known about the effect of noise on sleep quality in ICU patients. The study aim is to determine the effect of noise on subjective sleep quality.

**Methods:**

This was a multicenter observational study in six Dutch ICUs. Noise recording equipment was installed in 2–4 rooms per ICU. Adult patients were eligible for the study 48 h after ICU admission and were followed up to maximum of five nights in the ICU. Exclusion criteria were presence of delirium and/or inability to be assessed for sleep quality. Sleep was evaluated using the Richards Campbell Sleep Questionnaire (range 0–100 mm). Noise recordings were used for analysis of various auditory parameters, including the number and duration of restorative periods. Hierarchical mixed model regression analysis was used to determine associations between noise and sleep.

**Results:**

In total, 64 patients (68% male), mean age 63.9 (± 11.7) years and mean Acute Physiology And Chronic Health Evaluation (APACHE) II score 21.1 (± 7.1) were included. Average sleep quality score was 56 ± 24 mm. The mean of the 24-h average sound pressure levels (L_Aeq, 24h_) was 54.0 dBA (± 2.4). Mixed-effects regression analyses showed that background noise (*β* = − 0.51, *p* < 0.05) had a negative impact on sleep quality, whereas number of restorative periods (*β* = 0.53, *p* < 0.01) and female sex (*β* = 1.25, *p* < 0.01) were weakly but significantly correlated with sleep.

**Conclusions:**

Noise levels are negatively associated and restorative periods and female gender are positively associated with subjective sleep quality in ICU patients.

**Trial registration:**

www.ClinicalTrials.gov, NCT01826799. Registered on 9 April 2013.

**Electronic supplementary material:**

The online version of this article (10.1186/s13054-018-2182-y) contains supplementary material, which is available to authorized users.

## Background

Recently, hospital noise and its potential negative influence on patient outcome has gained widespread attention among caregivers [1, 2]. Noise is generally expressed as sound pressure in decibels (dB), whereby often a correction is made for the frequency of the sound (called “A-weighting”) to account for the relative loudness of the sound as perceived by the human ear. Noise levels in ICUs have been found to be beyond acceptable levels with average daytime sound pressure levels of around 60 A-weighted decibels (dBA) and peak levels > 90 dBA, the equivalent of standing next to a highway [3, 4]. Even more relevant, nighttime sound pressure levels are only slightly lower with averages of around 50 dBA. These sound pressure levels clearly exceed those of 35 dBA recommended by the World Health Organization (WHO) for nighttime in hospitals [5]. In the ICU, different factors contribute to high sound pressure levels, including a large number of alarm-generating monitoring equipment, use of mechanical ventilators and around-the-clock activities by staff members [4, 6, 7]. Excessive noise may cause multiple auditory and non-auditory effects, of which sleep disturbances are thought to be the most deleterious [8]. Sleep disturbances occur frequently in the ICU, characterized by an increase in stage 2 sleep and a decrease in stage 3 and rapid eye movement (REM) sleep [9, 10]. Up to now, only a few studies have studied the potential relationship between excessive noise and disturbed sleep in a real ICU setting. Studies using polysomnography in ICU patients demonstrated that between 11 and 24% of arousals are caused by environmental noise [11, 12]. Although polysomnography is considered the gold standard for evaluating sleep, it is labor intensive and a burden for ICU patients. Moreover, it is notoriously difficult to interpret and may not adequately reflect subjective sleep [13, 14]. Furthermore, studies have been performed in a small number of patients and in single centers, thus limiting the generalizability of the findings. Also, the acoustic parameters that have been analyzed are conventional measures of sound level, indicative of physical changes in the sound field. To further quantify the effects of noise on humans, advanced parameters, such as loudness and restorative periods, defined as periods of relative quietness, may be more useful. We therefore set up a prospective, multicenter observational study aimed to determine the association between various acoustic parameters and subjective perceived sleep quality in ICU patients.

## Methods

### Study design

This was a prospective observational multicenter study in the ICUs of five teaching hospitals and one university medical center in the Netherlands (CinicalTrials.gov. number NCT01826799). These hospitals were Jeroen Bosch Ziekenhuis,‘s Hertogenbosch (JBZ), Radboud University Medical Center, Nijmegen (RadboudUMC), Gelre Ziekenhuizen, Apeldoorn (Gelre), Isala Klinieken, Zwolle (Isala), Onze Lieve Vrouwe Gasthuis, Amsterdam (OLVG) and Ziekenhuis Gelderse Vallei, Ede (ZGV). Sound recording equipment was installed in 2–4 patient rooms in every participating ICU. Characteristics of the participating ICUs and rooms were collected, such as year of construction, layout, number of beds, level, population (e.g. surgical or cardiothoracic) and number of patients per room.

### Patients

ICU patients aged ≥ 18 years, admitted to one of the equipped rooms, were eligible after 48 h of admission to the ICU. Patients were not included if they were unable to understand Dutch or were unable to be assessed for sleep quality, defined as either a Richmond Agitation and Sedation Scale (RASS) score of − 2 or less or presence of delirium. Delirium detection was based on the Confusion Assessment Method for the ICU (CAM-ICU) three times daily.

Before study recruitment and enrolment, each patient and/or relative was given a full explanation of the study. Since this was an observational design and no actual sound recording was made inside the rooms that could be retraceable to individual patients, the need for informed consent was waived by the regional medical ethical committee (registration number MJ504, Medisch-Ethische Toetsing Onderzoek Patienten en Proefpersonen (METOPP), Tilburg, The Netherlands). Patient characteristics and demographics including relevant previous medical history, admission diagnosis, severity of illness score (expressed by the Acute Physiology And Chronic Health Evaluation (APACHE-)II score) and length of stay in the ICU were collected. Patients’ data were entered into a web-based electronic case record form by the research nurses of the participating hospitals and were only accessible by the investigators.

### Sound measurements

A measurement European conformity (CE)-marked microphone (M23, Earthworks Inc., Milford NH, USA) connected to a laptop or PC, was placed in 2–4 patient rooms, above the patients’ head (2.1~2.4 m from the floor). Only if the patient became eligible were the sound data used for analysis. The data were coded and stored on a hard disk and were de-identified to prevent identification of persons by members of the project group.

Based on previous research, the primary measures of interest for comparing the different acoustic conditions between the six hospitals were the A-weighted time-averaged sound pressure level (L_Aeq_) and the 10th percentile (L_90_) sound pressure level (A-weighted on fast-response mode), which is an estimate of background noise [4]. Furthermore, the occurrence rate of loudness peaks per hour, and the number and the average duration of restorative periods were recorded. A restorative period was defined as a continuous time interval of at least 5 min during which the sound pressure level (SPL) did not exceed the predefined threshold of 17.7 dBA above the L_90_ [4]. The threshold of 5 min was chosen to be the minimum time required for a patient to be able to go to sleep after a disturbance, and has been reported in the literature previously, while the 17.7 dB minimum relative sound level has been shown to be the average level needed to see an arousal in polysomnographic measurements of (healthy) persons exposed to ICU noise during sleep [15, 16].

The number of restorative periods per hour and their average duration were calculated. Longer restorative periods provide more opportunity for undisturbed sleep. The detection of loudness peaks was based on the psycho-physiological model by Chalupper and Fastl to ensure that the impact of peak sounds was assessed based on the auditory perception and expressed in units of sone [17]. Sone defines the sound level with respect to how the frequency sensitivity of the human ear changes with level, with us being less sensitive to lower frequency sounds at low sound levels, giving a metric that is perceptually more accurate yet harder to compute. A doubling of the sone level is equal to a doubling of the perceived loudness, unlike all of the other sound level measures reported in this paper, which are based on the logarithmic decibel scale. The rate was calculated either including all peaks or only peaks that had a minimum level of 10 sone, which is equivalent to a noise peak of at least 73 dB at 1 kHz. We calculated values for each parameter for three time periods: the whole day, day time (7 a.m.–11 p.m.), and night (11 p.m.–7 a.m.). Secondary measures of interest are presented and discussed in Additional file 1. Noise data were analyzed using Matlab version R2017a (The Mathworks Inc., Natick, MA, USA).

### Sleep assessment

Patients’ sleep was evaluated using the validated Richards Campbell sleep questionnaire (RCSQ) [18]. This 5-item questionnaire is used to evaluate different aspects of sleep, namely perceived sleep depth, sleep latency, number of awakenings, efficiency and time awake. Each item is rated on a visual analog scale (VAS) (0–100 mm), whereby higher scores indicate better sleep. The mean of the scores on these 5 items represents the overall RCSQ score. Usually, one item, regarding whether the noise level is disturbing for sleep is also part of the questionnaire [19, 20] and therefore this item was added to the questionnaire. The RCSQ has proven to be a valid, non-invasive tool for sleep perception in the ICU [18]. A Dutch translation of the RCSQ was created and validated according to the principles of good translation. Sleep evaluation was started after patients were identified as eligible, and was continued for a maximum period of five nights. The RCSQ was filled in by the patient at around 7 a.m.. If the patient was not able to fill in the RCSQ, no score was recorded.

### Statistical analysis

Data were compared using Student’s *t* test and proportions were compared using the chi-square test. To determine associations between variable noise parameters and sleep quality, exploratory hierarchical mixed-model regression analyses were performed specifying random intercepts for rooms in hospitals and for patients and selecting the best-fitting model using an automated model selection procedure based on the Akaike information criterion (AIC) [21, 22]. We based the calculation of *p* values on Satterthwaite estimated degrees of freedom and carried out the analyses on the data from the nighttime recordings between 11 p.m. and 7 a.m. The goodness of fit of the model was calculated based on the method described by Nakagawa and Schielzeth [23]. All statistical analyses were performed using SPSS version 20 (SPSS, IBM) and R (version 3.4.1, R Foundation for statistical computing, Vienna, Austria).

## Results

A total of 71 patients fulfilled the criteria between April 2013 and August 2015 and were included in this study. Data on seven patients were removed from the final analysis due to missing audio data. Baseline characteristics of the remaining 64 patients can be found in Table 1. On average, patients were 63.9 ± 11.7 years old and 48 (68%) patients were male. Most participating ICUs had single-bed rooms and the daily visiting routine occurred at similar times (see Table 2).

**Table 1:** Patient characteristics on inclusion; ^a^*n* = 62 due to missing data in 2 patients

Characteristic	Patient cohort (*n* = 64)
Patient characteristics	
Male sex, (*n* (%))	44 (69)
Age (mean, SD)	63.9 (11.7)
APACHE II score (mean, SD)	21.1 (7.1)
SOFA score (mean, SD)	6.1 (3.7)
ICU admission duration before inclusion (median days, IQR)	4 [3–10]
Admission category	
Respiratory	15 (23.4)
Cardiology	2 (3.1)
Medical	19 (29.7)
Neurology	1 (1.6)
Surgery	27 (42.2)
Prior history	
Cognitive dysfunction (*n* (%))	4 (6.3)
Delirium during ICU stay (*n* (%))	12 (19.4)^a^
Hearing problems (*n* (%))	2 (3.1)
Alcohol abuse (*n* (%))	7 (10.9)
Current status	
Use of sedatives (*n* (%))	11 (17.2)
Isolation measures (*n* (%))	17 (26.6)
Ability to speak (*n* (%))	47 (73.4)
Invasive mechanical ventilation (*n* (%))	20 (31.2)
Mechanical ventilation duration (median days, *n* IQR)	12 (9–23)

*APACHE* Acute Physiology And Chronic Health Evaluation, *SOFA* Sequential Organ Failure Assessment

**Table 2:** Hospital characteristics

Hospital	Site 1	Site 2	Site 3	Site 4	Site 5	Site 6
Patients, *n* (%)	16 (25.0)	5 (7.8)	13 (20.3)	9 (14.1)	14 (21.9)	7 (10.9)
Year of ICU construction	2008	2013	2011	2003	2011	2000
Type of ICU	Medical Surgical	Medical Surgical Cardiothoracic	Medical Surgical	Medical Surgical Cardiothoracic	Medical Surgical Neurosurgical Cardiothoracic	Medical Surgical
Number of beds	14	36	14	24	40	12
Number of beds per room	1	1	1	1 to 2-4	1	1
Nursing handovers, h	0700–1500–2300	0730–1530–2315	0730–1530–2300	0730–1500–2315	0700–1500–2300	0730–1500–2245
Medical handovers	Not in patient rooms	0830–1700–2330	0800–1630–2300	0845–1500–2315	1000–1600–2300	Not in patient rooms

### Sleep quality of the patients

No sleep evaluation was registered for five patients; finally, 151 nights of sleep were evaluated (mean 2.4 nights/patient). Average total sleep quality was 56 ± 24 mm and was not significantly different between the participating hospitals (Table 3). Based on the additional question of the RCSQ, noise was considered quite disturbing with an average VAS score of 34 mm, whereby a lower score indicates more disturbance. In 64 of 151 nights (42%), patients provided an answer to which noise they found was the most disturbing factor during their sleep. Patients found that monitor/equipment alarms were the most disturbing to sleep (28/64) followed by other (*n* = 21), staff speech (*n* = 9), and other staff activities (*n* = 6).

**Table 3:** Results from the sleep evaluation per site and of all sites; average scores per item of the Richards Campbell sleep questionnaire (RSCQ) and the average overall RCSQ score are expressed as mean mm (SD)

Site number	Sleep depth	Falling asleep	Awakening	Return to sleep	Sleep quality	Average RCSQ score
1	50 (26)	67 (31)	59 (26)	52 (30)	59 (27)	57 (23)
2	64 (24)	54 (33)	66 (30)	56 (26)	69 (26)	62 (26)
3	52 (26)	44 (29)	50 (25)	48 (34)	46 (31)	48 (23)
4	46 (16)	56 (24)	49 (24)	50 (27)	51 (28)	50 (20)
5	61 (28)	64 (31)	65 (26)	59 (32)	60 (30)	62 (27)
6	60 (16)	67 (22)	61 (21)	56 (22)	65 (22)	62 (18)
All sites	54 (25)	60 (30)	58 (26)	53 (30)	57 (29)	56 (24)

### Noise levels in participating ICUs

**Fig. 1:**
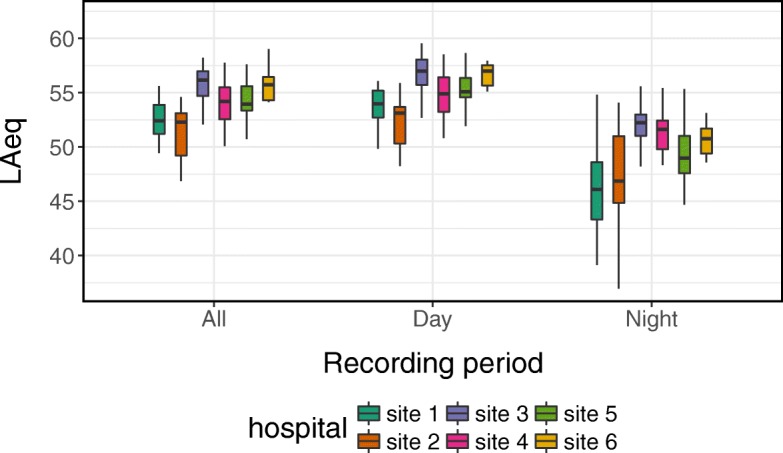
The 24-h average sound pressure levels (L_Aeq,24h_) for three recording periods: all (24 h, midnight to midnight); day (from 7 a.m. to 11 p.m.); night (from 11 p.m. to 7 a.m.)

The mean of the 24-h average sound pressure level (L_Aeq, 24h_) was 54.0 ± 2.4 dBA, with no significant differences between day and night (see Table 4 and Fig. 1). The L_90_ was 38.1 ± 4.0 dBA on average. Restorative periods occurred on average 1.2 times per hour during the day, increased to 2.4 during the night (*p* < 0.0001), whereby also the average duration of the restorative period significantly increased from 11.4 min during the day to 14.1 min during the night (*p* < 0.001, see Table 4). Loudness peaks with a minimum magnitude of 10 sone occurred 23.1 times per hour during the day, significantly decreasing to 6.0 times per hour during the night (*p* < 0.0001).

**Table 4:** Averages (SD) for five selected noise level parameters for each hospital individually (nighttime) and averages for all hospitals during 24 h, day time (7am - 11pm), and night (11pm - 7am)

Auditory parameter	Site 1	Site 2	Site 3	Site 4	Site 5	Site 6	Average		
							24 h	Day	Night
L_Aeq_ (dBA, SD)	46.3 (3.9)	47.1 (5.8)	51.7 (3.2)	51.5 (2.2)	49.8 (2.7)	50.7 (1.5)	54.0 (2.4)	55.1 (2.3)	49.2 (4.0)
L_90_ (dBA, SD)	35.9 (4.2)	37.8 (1.9)	42.0 (3.6)	43.6 (1.9)	36.2 (4.0)	40.0 (2.7)	38.1 (4.0)	39.1 (3.9)	38.4 (4.7)
Peaks10S (count, SD)	2.5 (3.3)	4.1 (4.3)	8.4 (7.0)	8.8 (6.2)	7.2 (6.6)	7.2 (4.1)	17.5 (8.0)	23.1 (10.1)	6.0 (6.1)
Average number of restorative periods (count, SD)	20.5 (5.9)	15.9 (8.6)	19.5 (4.8)	18.4 (5.4)	17.9 (7.7)	19.6 (6.2)	38.3 (12.5)	19.4 (9.5)	19.1 (6.4)
Average durations of restorative periods (min, SD)	12.6 (5.5)	11.6 (5.5)	17.6 (7.9)	10.6 (2.1)	15.1 (10.5)	13.8 (5.5)	12.9 (7.7)	11.4 (9.4)	14.1 (7.7)

L_Aeq_ A-weighted time-averaged sound pressure level, *dBA* A-weighted decibel in ICU, *L*_*90*_ 10th percentile sound pressure level (A-weighted on fast-response mode), *Peak10S* hourly rate of loudness peaks of at least 10 sone

### Association between noise and sleep

**Fig. 2:**
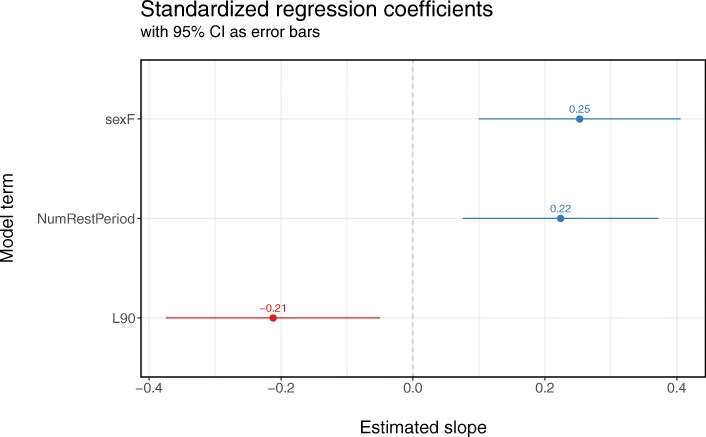
Standardized regression coefficients for the best fitting model for sleep quality evaluated by the patients. Error bars indicate 95% confidence intervals. Standardized coefficients are expressed in units of standard deviations to enable easy assessment of which of the regression predictors imparts the largest changes in the regression outcome variable

Female patients rated their sleep quality on average 1.2 points higher than men (*p* < 0.01; see Fig. 2 and Additional file 1). The number of restorative periods per hour during the night also significantly positively contributed to sleep quality: with every additional restorative period (per hour), sleep quality significantly increased by 0.53 points (*p* < 0.01). Higher levels of the background noise (L_90_) significantly decreased sleep quality ratings by 0.51 points (*p* < 0.05) (Fig. 2). We identified conditional ${R}_{glmm(c)}^2$ of 0.2; regression model diagnostics did not highlight violations of model assumptions. Note that we did not incorporate the additional RCSQ item, regarding which noise source was most disturbing for sleep, as the predictor or outcome variable in the regression analysis because we did not gather a sufficient number of data points to enable us to make reliable conclusions. A more elaborate description of the analyses and model diagnostics is provided in Additional file 1.

## Discussion

In this prospective multicenter, observational study, we showed that background noise was negatively associated with sleep, while gender (female) and the number of restorative periods were positively correlated with sleep quality. Patients’ perceived sleep quality was poor and did not differ between participating hospitals. Overall, noise levels were consistently above the values (< 35 dB L_Aeq_ day and night; < 40 dB L_AFmax_ night) recommended by the WHO [5]. This is the first study to evaluate a link between subjective sleep quality and objective noise parameters in ICUs.

Sleep disturbances are very common in ICU patients [13]. As a risk factor for sleep disturbances [13], noise in ICUs is ubiquitous and is mainly caused by staff activity, machines and alarms [4].

Research on the relationship between noise and sleep arousals in ICU patients, thereby using polysomnography, shows that noise peaks > 80 dBA are associated with arousal from sleep and that noise is responsible for 11 to 24% of the total number of arousals [11, 12, 24]. More subjectively, patients themselves consider noise in the ICU to be disturbing to sleep [25].

We found that a higher L_90_ led to a moderate decrease in sleep quality. L_90_ is considered to be indicative of the background noise level, generated by, for example, air conditioning or computer ventilators [26]. The average nighttime value of L_90_ was 38.1 dBA, indicating that levels of background noise even exceed the threshold for average sound pressure levels stated by the WHO. Because the participating hospitals were different in design and layout, considerable differences were found in L_90_ between hospitals. Given that within-hospital L_90_ was not different between day and night, the observed differences cannot be attributed to differences in procedures and staff movement but must be due to the building characteristics. This finding underlines the importance of taking building properties into account when designing a new ICU. Average day and nighttime noise levels were comparable with others studies, whereby differences between day and night were only marginal [3, 4].

This is the first study showing a positive association between restorative periods and better sleep. Restorative periods occurred on average only 2.4 times per hour during the night, and the average duration of a restorative period was 14.1 min, which is indicative of the high number of peak noises. Since restorative periods are most frequently ended by high-level noisy events due to staff activity or speech [4], interventions aimed at reducing staff-generated noise appears to be a reasonable and achievable goal to improve sleep quality in critically ill patients. Additionally, since noise coming from alarms and monitors were found to be the most disturbing noise sources in our qualitative analysis, nighttime alarm modification may also be of additional value in improving sleep quality in critically ill patients.

Female patients expressed that they had better sleep compared to the male patients. Gender differences in subjective sleep quality in ICU patients have not been reported previously in the literature, however, studies in the general population generally indicate worse subjective sleep quality in women, compared to men [27]. Interestingly, in a large study on the effects of traffic noise on objective sleep and subjective sleep quality in healthy people, there were larger effects of noise on objective sleep parameters in men compared to women, whereas no clear differences were found in subjective sleep quality [28]. Future research on gender differences in sleep quality should further elucidate this finding.

In addition to pure physical properties of sound, we also analyzed the impact of noise peaks on human perception (also called loudness) by using a validated model [17]. Loudness peaks with a magnitude of at least 10 sone occurred significantly less during the night than during the day. It is noteworthy that the nighttime hourly rate of these peaks differed substantially between the participating hospitals, varying from 2.5 to 8.8. Without a source-specific analysis, it is difficult to pinpoint the source of these loud peaks and what causes this difference between participating hospitals.

Some limitations need to be addressed. First, we used the RCSQ as a measure of sleep quality instead of polysomnography. Although RCSQ is validated for the use with ICU patients, subjective evaluation of sleep quality is subject to different forms of bias, including recollection and response bias, which may specifically be true for ICU patients who are recovering from critical illness. However, the results of the sleep evaluations are in line with previous studies and the repetitive design of up to five measurements helps in reducing the impact of outliers. Moreover, the RCSQ is easily applicable and interpretable and is therefore easier to use in daily practice in contrast to polysomnography. Second, we included a relatively small group of ICU patients who were awake and able to communicate, thus selecting only a subgroup of less severely ill patients, which makes it difficult to generalize the findings of this study to the whole ICU population. Although sleep evaluation in sedated and/or delirious patients remains difficult, interventions aimed at improving sleep quality by addressing noise may also have beneficial effects in this other patient group. Third, we did not take the long-term outcomes of the patients into account. Therefore, negative effects on sleep may not necessarily lead to worse outcomes. However, other studies have clearly demonstrated an association between sleep deprivation and the development of delirium in ICU patients, which has a multitude of negative long-term consequences [29]. Moreover, even brief periods of sleep deprivation in the general population can have long-term negative consequences in immune and cognitive function and in hypertension and obesity [30].

## Conclusions

Associations between various noise parameters and subjective sleep quality were found in this multicenter study, confirming the negative consequences of noise on the sleep quality in ICU patients and thereby strengthening the usefulness of noise-reducing strategies. Sleep quality in general was poor and did not differ between participating ICUs. Noise levels were high and periods of relative quietness occurred only rarely. Increasing the number of nighttime restorative periods appears to be a reasonable goal for improving patients’ sleep.

## Additional file

Additional file 1: Detailed description of acoustical analysis and model selection. (PDF 164 kb)

## Abbreviations

AIC: Akaike information criterion
APACHE: Acute Physiology And Chronic Health Evaluation
dB: Decibel
dBA: A-weighted decibel Intensive Care Unit
L_90_: 10th Percentile sound pressure level (A-weighted on fast-response mode)
L_Aeq_: A-weighted time-averaged sound pressure level
L_Aeq, 24h_: A-weighted time-averaged sound pressure level over 24 h
L_AFmax_: A-weighted sound pressure level on fast mode
METOPP: Medisch-ethische toetsing onderzoek bij patiënten en proefpersonen (regional medical ethical committee)
RASS: Richmond Agitation and Sedation Scale
RCSQ: Richards Campbell sleep questionnaire
REM: Rapid eye movement
SPL: Sound pressure level
VAS: Visual analog scale
WHO: World Health Organization
