# Supplementary material for: Noise in the intensive care unit and its influence on sleep quality: a multicenter observational study in Dutch intensive care units
Source: Crit Care. 2018 Oct 5;22:250. doi: 10.1186/s13054-018-2182-y (PMC6173893; doi:10.1186/s13054-018-2182-y)
Supplement: Supplementary file 1 — Detailed description of acoustical analysis and model selection. (PDF 164 kb) [file 13054_2018_2182_MOESM1_ESM.pdf]

Noise in the intensive care unit and its influence on sleep quality: a multicenter observational study in Dutch intensive care units

Supplementary material

Koen. S. Simons, Eva Verweij, Paul M. C. Lemmens, Sam Jelfs, Mun Hum Park, Peter E. Spronk, Johannes P. C. Sonneveld, Hester M. Feijen, Marijke S. van der Steen, Armin G. Kohlrausch, Mark van den Boogaard, & Cornelis P. C. de Jager

Contents

1 Acoustic parameters **1**

2 Hierarchical / mixed-models regression **4**

2.1 Model selection procedure . . . 5

2.2 Model fit and diagnostics . . . 5

2.3 Influential data . . . 6

2.4 Combined effect of acoustic predictors . . . 8

2.5 Individual RCSQ items . . . 8

References **9**

1 Acoustic parameters

In the main paper we focused on five acoustic parameters as having primary interest: average sound–pressure level (L_{Aeq}), background–noise level (L_{90}), the rate of loud peaks per hour with a magnitude of at least 10 sone (LoudPeaks10S), and the number and duration of the restorative periods (NumRestPeriod and AvgDurRestPeriod). In addition to these parameters, we calculated an additional series of parameters that we discuss and present here. Collectively, these parameters enabled a more complete assessment of the acoustic environment of the participating ICUs. Note that for the visualizations below, we used the untransformed data and removed one outlier value for the average duration of restorative period.

Figures 1 – 5 provide an overview of the distribution of data (using box and whisker plots) from all calculated parameters. Figure 1 (left-hand panel) illustrates that hospitals were fairly similar[1] in terms of average sound–pressure level (L_{Aeq}) and shows that, on average, sound–pressure level (SPL) dropped about 6 dBA from 55 dBA during the day to 49 dBA during the night which may be a relevant reduction given the fact that an approximately 10 dB decrease equates to an approximately halving of loudness. Those averages are, however, higher than the WHO recommendations for average nighttime sound–pressure levels in hospitals (35 dBA) [1]; In fact, even the average 24-hour background–noise level of 38.1 dBA (L_{90}; see Table 3 in the main article) is higher than the recommended

[1]Note that we deliberately did not test whether the differences between hospitals were statistically significant because, first and foremost, the observational nature of the present study precluded reliably explaining these differences due to the many confounded or interacting differences between hospitals. We were also worried that we would make too many false discoveries (even after performing multiplicity corrections) due to the large number of comparisons we would need to make.

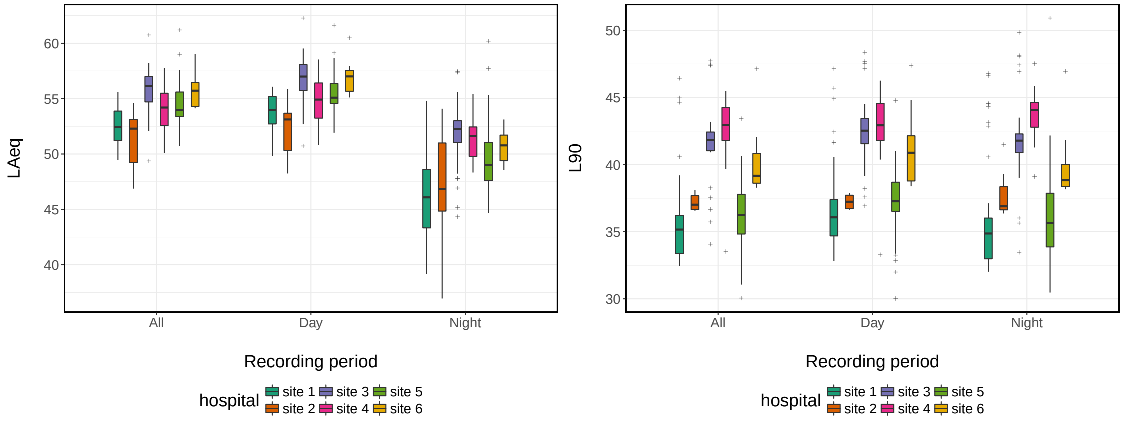

Figure 1: Box and whiskers plot for average and background (A-weighted, fast) sound–pressure level (L_{Aeq} and L_{90}, respectively) for each hospital and three recording periods. Recording period All: 0:00 - 23:59; Day: 7:00-22:59; Night: 23:00 - 7:00 with 7:00 being the moment when an RCSQ was completed.

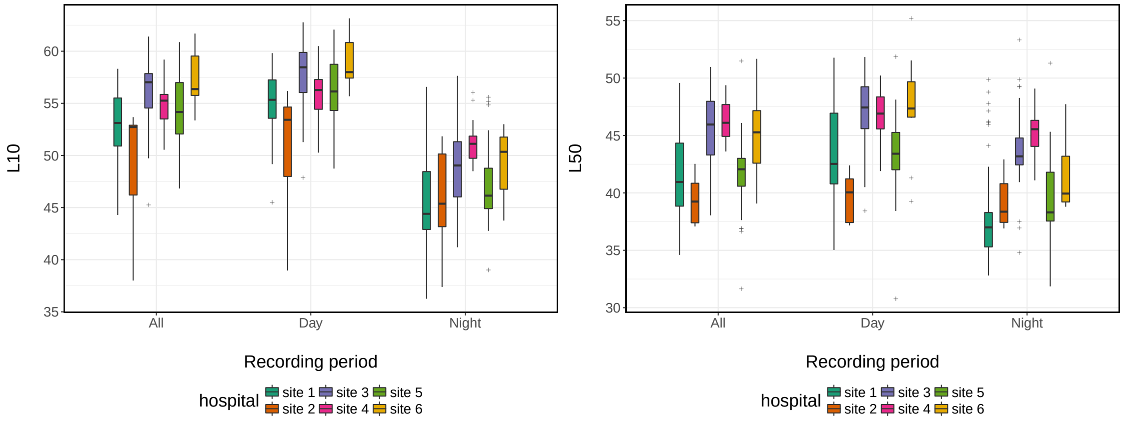

Figure 2: Box and whiskers plot for the 90th percentile and median (A-weighted, fast) sound–pressure level (L_{10} and L_{50}, respectively) for each hospital and three recording periods. See Figure 1 for details on the recording periods.

threshold of 35 dBA. The right–hand panel shows that, when comparing time periods within hospitals, the distributions hardly changed, implying that L_{90} truly reflects background–noise levels due to, for instance, building characteristics like type of air conditioning.

Figure 2 completes the data regarding the distribution of sound–pressure levels showing box and whiskers plots for the 90th and 50th (median) decile (L_{10} and L_{50}, respectively). Adding the right-hand panel of Figure 1 to the right of these two panels illustrates how the distributions of sound–pressure levels (SPL) changed from having a wider shape and, within hospitals, considerable differences between day and night for L_{10}, to very narrow distributions for L_{90} with no discernable differences between SPL comparing day and night. This highlights that background noise L_{90} is, as expected, fairly stable during the day (within a hospital) but that “foreground” sound (L_{10}) varies in SPL to a greater extent and also that it, again expected, reduces during the night. The shapes and placement of the distributions of L_{50} (Figure 2, right–hand panel), representing the middle/median values, nicely capture the mid point between L_{10} and L_{90}.

The next interesting comparison is regarding peak noises. Figure 3 shows in the left-hand panel the number of loud peaks with a magnitude of at least 10 sone whereas the right-hand panel shows the number of loud peaks irrespective of magnitude. Obviously, the total number of loud peaks per hour is higher when including all peaks (right-hand panel) but more interesting is that during the night, compared to the total number of sound peaks,

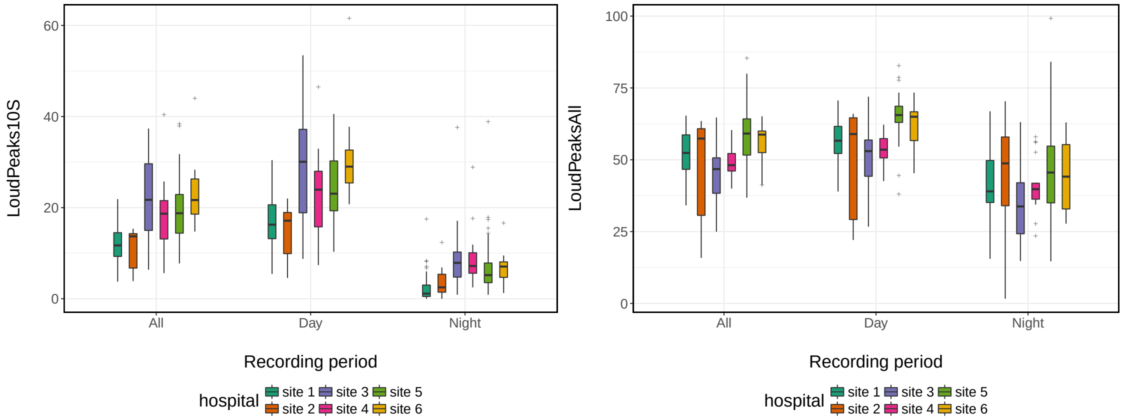

Figure 3: Box and whiskers plot for the rate of loud peaks (per hour) with a 10 sone magnitude (left-hand panel) and all peaks (independent of magnitude; right-hand panel). See Figure 1 for details on the recording periods.

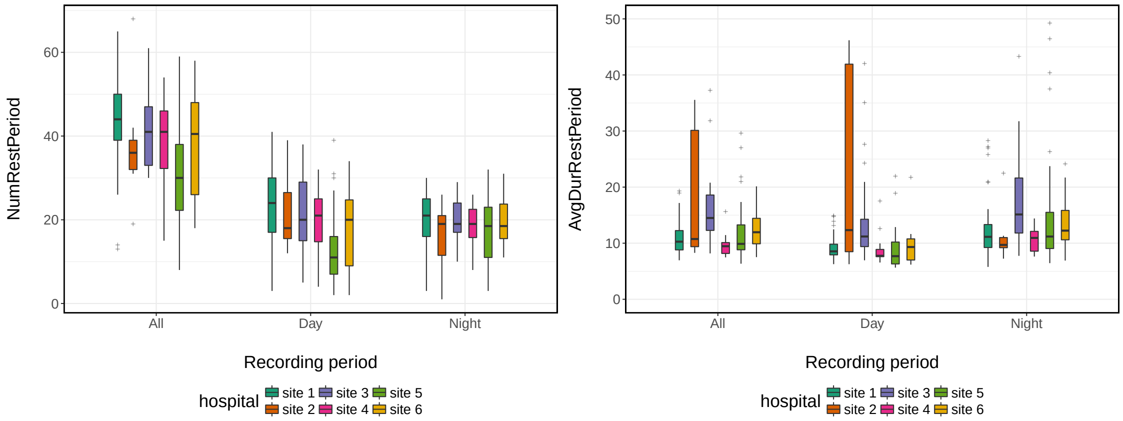

Figure 4: Box and whiskers plots for the number of (left panel) and average duration of the restorative periods (in minutes; right-hand panel) for the three recording periods (see Figure 1). Note that the range of number of restorative periods is different between the three time periods due to having different durations. For average duration of restorative periods, we zoomed in on data between 0 and 50; this removed two data points at around 55 and 70 minutes.

the number of peaks exceeding 10 sone decreased to a larger extent. Given that the sound-pressure level (L_{90}) did not change from day to night, and that the loudness of the alarms (presumably) also does not change, the larger reduction in number of 10 sone peaks during the night is most likely due to a reduction of staff and/or visitors activity in the ICU.

The remaining parameters, that we did not discuss yet, are the number and duration of the restorative periods (see Figure 4). The relationship between these two parameters is complicated in that compared to fewer and shorter restorative periods, more and longer is better for recovery, but more restorative periods also implies that (logically) their average duration needs to be shorter. It might be surprising that the distributions of number of restorative periods during day and night seems similar. However, considering that the day period comprises a 16-hour period and night only 8 hours, it is clear that during the night, more restorative periods occur and, at least visually (see Footnote 1), they seem to have a slightly longer duration. Combining the previous observation regarding peak sounds with the present one, we therefore suggest, in the main paper, that interventions to reduce staff-generated noise seems to be a reasonable and achievable goal to try and improve sleep quality of patients in ICUs.

We also assessed the maximum sound–pressure level of peak sounds per minute (indexed using the $L_{AFmax,1min}$ parameter; see Figure 5), which is also related to short-time high-level sound events (peak sounds) but now based on

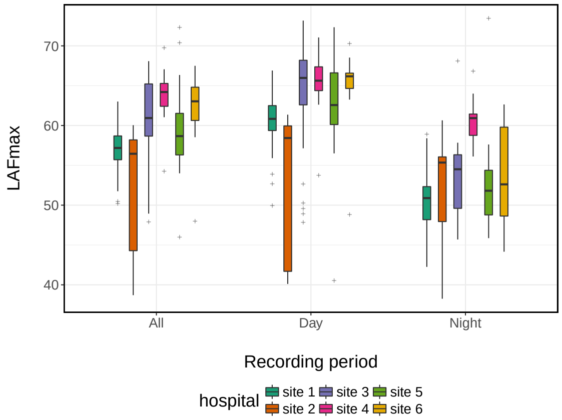

Figure 5: Box and whiskers plots for the average of the maximum sound–pressure level per minute ($L_{AFmax,1min}$) for the recording periods that are explained in the caption of Figure 1.

a physics model whereas the peak sounds in Figure 3 are based on a psychophysiological model [2]. We noted that the average level of $L_{AFmax,1min}$ is lower during the night than during the day, but that it exceeds the recommended noise level of 40 dBA that is proposed by the WHO (Table 4.1) [1]. It is these peak sounds that typically end a restorative period. Note again that Figure 3 shows a perception–based perspective on peak sounds whereas Figure 5 takes an acoustic (physics) view using sound–pressure levels. It is clear that the sound–pressure level of some of those peak sounds cannot be reduced because they are caused by alarms that the staff need to hear. The observational nature of the study precludes concluding that only alarms causes these excessive SPL's or that also staff activity increases SPL's in the ICU and, if so, whether specific activities cause them.

2 Hierarchical / mixed-models regression

Hierarchical (mixed) modeling is a regression technique that exploits intra-class clustering of data to generate more precise estimates of the fixed-effects parameters of a regression and, additionally, uses this information to calculate the variance around the fixed-effects parameters that each cluster introduces in the data [3].

The present data set contained two sources of variation: patients that are randomly included in the study and hospital rooms that have unique properties that may affect sleep quality ratings of the patients in ways that should be prevented from affecting the analysis. We therefore specified a random effects structure that contained a random intercept for rooms within hospitals and a random intercept for patients. Note that preliminary analyses highlighted that a random intercept for hospitals was not necessary for an adequate model fit.

We transformed the data to grand-mean centered and scaled scores for all acoustic variables because some of those were on different magnitudes of scales. Moreover, scaling also reduces effects of multicollinearity. We used R (version 3.4.1; [4]) and lme4 (version 1.1-14) for the hierarchical mixed-models regressions. Model selection was supported by the glmulti package (version 1.0.7) and least-squares means were calculated using the effects package (version 3.1-2). For the calculation of R^2_{glmm} based on Nakagawa and Schielzeth [5], we used the piecewiseSEM package (version 1.2.1). Existence of influential data was assessed using the influence.ME package (version 0.9-9; [6])

Figure 6 shows, as an example, the relationship between sleep quality determined using the RCSQ and one of the selected acoustic parameters: A-weighted sound–pressure level (L_{Aeq}). The scatter plot shows considerable variability in RCSQ scores: that is, patients indicated bad sleep quality for low average SPLs as well as for high average SPLs.

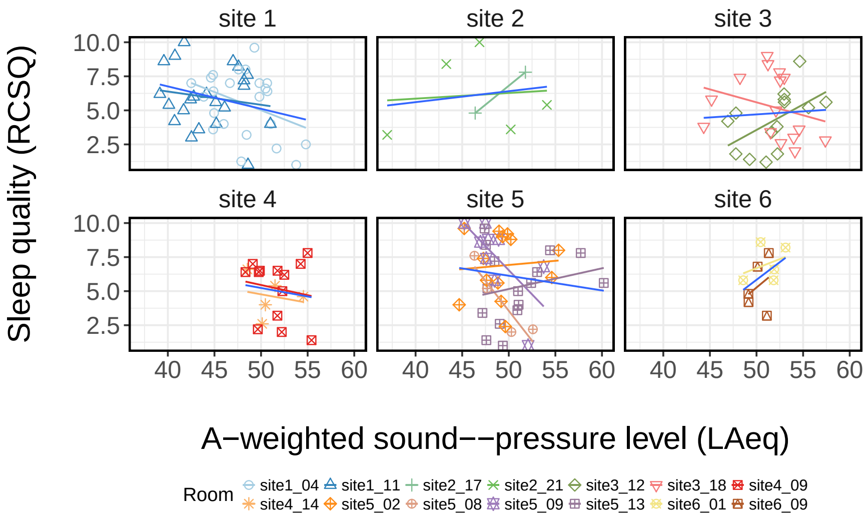

Figure 6: An example scatter plot of L_{Aeq} against sleep quality for the sleep quality measurements obtained for the individual rooms (with hospitals in separate panels) to highlight the variability in sleep quality scores measured using the RCSQ. Colors and shape indicate individual rooms with simple regression lines for each room in the same color; the blue regression line is the overall regression for the respective hospital. The scatter plot is based on untransformed data.

2.1 Model selection procedure

The current exploratory modeling approach is different from a confirmatory study where theory dictates which parameters are important because in the present study we explored which parameters were relevant rather than confirming that specific parameters affected sleep quality. We therefore worked from the assumption that the best fitting statistical model would have the most explanatory power to indicate, in the present data set, which acoustic parameters most strongly affected sleep quality. This approach typically requires that a subset of relevant parameters is selected from a larger set of candidate parameters.

In regression analyses this selection is often performed based on stepwise backward regression by fitting a full model and then, one by one, eliminating non-significant predictors. This method, however, is known to be fraught with problems [7]. The most relevant problems are that the stepwise regression inflates R^2 values and yields confidence intervals that are falsely narrow and affect p-values negatively [7], and that the procedure does not necessarily find the best fitting model [8].

We therefore chose to use a model-selection procedure based on minimizing the (sample size corrected) Akaike information criterion (AICc) [8–10] to find the model with the least amount of parameters having the best explanatory value as indicated by the lowest AICc. The resulting model is presented in the main body of the article and below we elaborate on model fit and diagnostics.

2.2 Model fit and diagnostics

In normal, simple or multiple, regression, model fit is assessed using (adjusted) R^2 which is a value that can be interpreted as the percentage of explained variance. For mixed-effects regression such an assessment of model fit is more difficult because it can be implemented in a number of ways and is known to have theoretical problems [5]. Based on a series of requirements for an ideal R^2 score, Nakagawa and Schielzeth [5] therefore proposed two R^2 values applicable to (generalized) linear mixed models: the marginal R^2 that reflects explained variance based only on the fixed effects of a mixed-effects regression, and the conditional R^2 that incorporates the entire model in its score.

Table 1: Model summary of the best-fitting model (based on minimizing the AICc) for the patients' RCSQ scores including the random effects.

Model term	Estimate	SE	t statistic	95% CI
Intercept	5.209	0.257	20.294	[4.7, 5.7]
sexF	1.247	0.384	3.246	[0.5, 2]
L90	-0.505	0.197	-2.559	[-0.9, -0.1]
NumRestPeriod	0.533	0.180	2.964	[0.2, 0.9]
Patients	0.260			
**Random intercept**				
Rooms	0.399			
Residual error	2.149			

For the model that we selected as best model, these scores were $R^2_{glmm(m)} = 0.1$ and $R^2_{glmm(c)} = 0.2$. These scores indicated that the model did not explain a lot of the variance in the data. The relatively low $R^2_{glmm(c)}$ reflect that variance remained in the data that was not explained by fixed or random effects. This implies that other factors may have affected patients' sleep scores that we have not yet considered. We further evaluated model fit using the common-place residual plots (see Figure 7). The visual analysis did not highlighted problems with the assumptions of the regression model.

Note that model-selection procedures based on information criteria typically result in a set of candidate best models that should be evaluated for their relative merit(s) based on theoretical and statistical grounds. The present analysis indeed proposed a model involving the SOFA score as additional predictor as best fitting model with the lowest AICc. The estimated slope for the SOFA score, however, was not significant and the relative improvement in model fit was not significant either (as determined using a Loglikelihood Ratio Test; $p > 0.05$). Therefore, we decided to remove the SOFA score from the set of selected predictors despite its theoretical relevance. The theoretical relevance arises from earlier research that has shown that disease severity can be related to noise disturbances due to, for instance, increased alarms and activity around a more severely ill patient [11]. The decision to remove the SOFA score from the model left gender as the only patient-related parameter that influenced sleep quality.

2.3 Influential data

Finally, we verified whether specific data points, for instance, data from a specific patient or a specific room, had excessive influence on the regression estimation by having data points that were too close or too far away from the estimated regression equation. For mixed-effects models, determining influence and leverage is, again, more complicated than for normal (simple or multiple) regression because when calculating values like dfbetas or Cook's distances [12, 13] we needed to consider that data points were part of a group of data points that belong to a room or a patient. Nieuwenhuis and colleagues [6] developed an R package that enables calculating these influence and leverage scores using a leave-one-out approach on pre-specified levels of analysis.

For the (hierarchical) level of patients, we found that data of two participants was often flagged as having passed the cutoff value for the various scores (e.g., see Figure 8 for a visualization of Cook's distance). When we removed the data from that participant and refitted the model, we observed that all slopes in the model reduced in size (to 1.08, -0.43, and 0.38 for females, L_{90}, and number of restful periods, respectively) but did not change in significance. We therefore decided to keep these participants included the data. For the level of rooms, we found similar changes in the slopes that, again, did not affect significance.

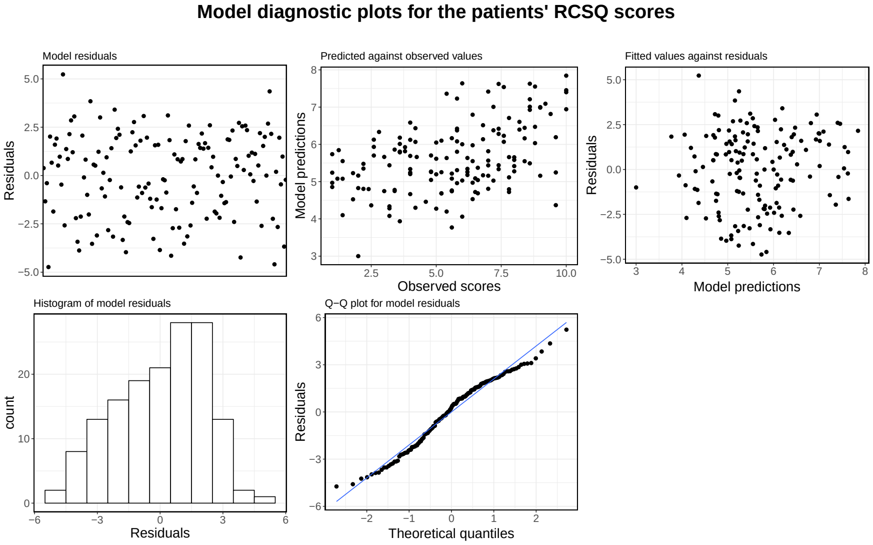

Figure 7: A series of model-diagnostic plots on the model residuals illustrate (top left to bottom right) a random distribution of the residual error terms, the model fit, no discernable relation between fitted values and residual, and normally distributed residuals evidenced by a Q-Q plot and a histogram.

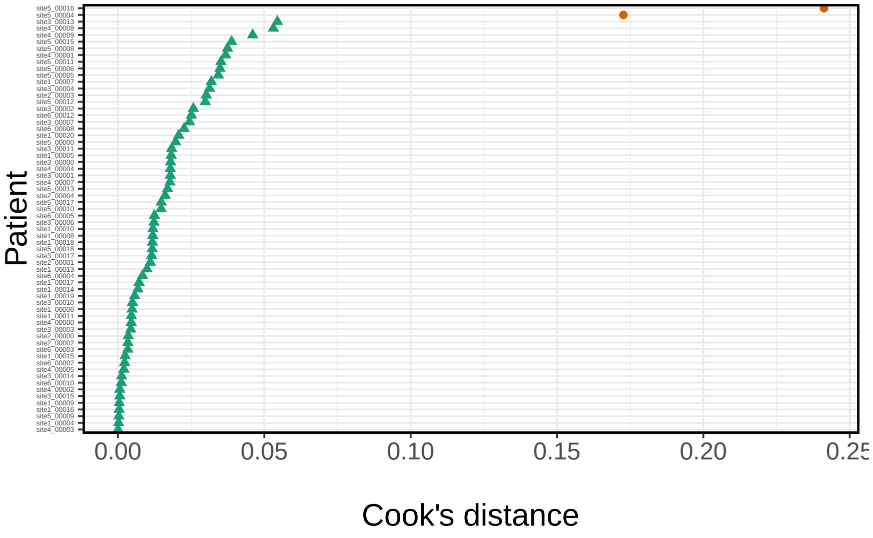

Figure 8: Cook's distance calculated for individual patients for all variables in the selected regressione equation. Data points highlighted in red triangles indicate data points with excessive influence on the fit of the model using the conventional criterion of 4 divided by the sample size.

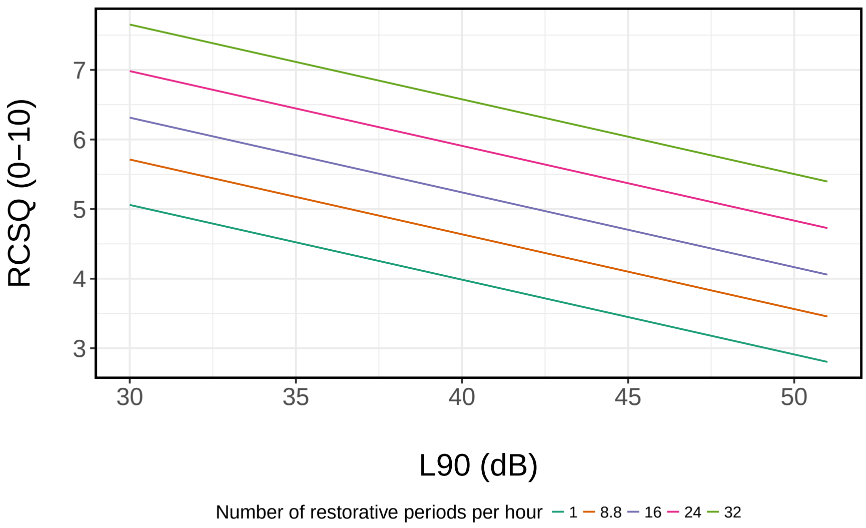

Figure 9: Overview of the combined effect on sleep quality of the acoustic predictors in the regression model. Note that the data are visualized in back transformed values. The values for number of restorative periods are arbitrarily chosen values that represent the entire spectrum of values observed in the data.

2.4 Combined effect of acoustic predictors

For improved understanding of the combined effect on sleep quality of the acoustic predictors L_{90} and number of restorative periods, we created a marginal effects plot that displays how (predicted) sleep quality would change as a result of changes in value of the acoustic predictors (while averaging over the other predictors). Figure 9 shows the predicted sleep quality for various ranges of background sound (L_{90} on the *x*-axis) and numbers of restorative periods (in separate lines). This figure shows that under the best circumstances (i.e., many restorative periods and low background-noise levels), sleep quality was estimated at approximately 7.3 and the worst sleep quality is predicted for a low rate of restorative periods and high background noise.

2.5 Individual RCSQ items

The above analyses as well as those in the main text were performed on the overall score of the RCSQ based on the average of the items' scores. However, the items of the RCSQ were designed in such a way that they correlate well with specific sleep stages and/or aspects of sleep. That is, items 1 - 5 gather scores on the following aspects of sleep, respectively: sleep depth, falling asleep, number of awakenings, percent of time awake, and quality of sleep.

It could be that the scores regarding each of these aspects were affected by different parameters. We therefore ran an automated-selection procedure on each of the individual items' data to determine which of the available parameters were selected as part of the best model for an item. Most model-selection procedures resulted in a best model that incorporated the same set of parameters as the main model presented above. We therefore only verbally discuss the exceptions and conclude that in the present data the overall RCSQ score correlated well with the scores for its items.

Newly introduced parameters were all based on patients' data, for instance, whether or not a patient was on ventilator support (relevant for number of awakenings and quality of sleep), duration of stay in hospital before ICU admittance (relevant for sleep depth and number of awakenings), and duration of the stay in the ICU (for percentage of time awake). Being on invasive ventilator resulted in significantly fewer awakenings during the night whereas quality of sleep was significantly negatively affected by being on a non-invasive ventilator.

We therefore concluded that the overall RCSQ was well supported by the scores on the individual items. That is, the statistical model for the total score mostly comprised the same parameters as the models for the individual items

rather than that the items' models contained many other parameters.

References

1. Berglund B, Lindvall T, H. SD, WHO Occupational and Environmental Team (1999) Guidelines for community noise. Geneva : World Health Organization

2. Chalupper J, Fastl H (2002) Dynamic loudness model (DLM) for normal and hearing-impaired listeners. Accessed 18 May 2018

3. Gelman A, Hill J (2007) Data analysis using regression and multilevel/hierarchical models. Cambridge University Press, Cambridge ; New York

4. R Core Team (2017) R: A language and environment for statistical computing. R Foundation for Statistical Computing, Vienna, Austria

5. Nakagawa S, Schielzeth H (2013) A general and simple method for obtaining r^2 from generalized linear mixed-effects models. Methods in Ecology and Evolution 4:133–142. doi: 10.1111/j.2041-210x.2012.00261.x

6. Nieuwenhuis R, Grotenhuis M te, Pelzer BJ (2012) Influence.ME: Tools for detecting influential data in mixed effects models. The R Journal 4:38–47

7. Harrell Jr. FE (2015) Regression modeling strategies with applications to linear models, logistic and ordinal regression, and survival analysis. Springer International Publishing :

8. Calcagno V, Mazancourt C de (2010) Glmulti: An r package for easy automated model selection with (generalized) linear models. Journal of Statistical Software 34:

9. Greven S, Kneib T (2010) On the behaviour of marginal and conditional AIC in linear mixed models. Biometrika 97:773–789. doi: 10.1093/biomet/asq042

10. Wagenmakers E-J, Farrell S (2004) AIC model selection using akaike weights. Psychonomic Bulletin & Review 11:192–196. doi: 10.3758/BF03206482

11. Park M, Vos P, Vlaskamp BN, et al (2015) The influence of APACHE II score on the average noise level in an intensive care unit: An observational study. BMC Anesthesiology 15:42. doi: 10.1186/s12871-015-0019-7

12. Belsley DA, Kuh E, Welsch RE (2004) Regression diagnostics: Identifying influential data and sources of collinearity. Wiley, Hoboken, N.J.

13. Cook RD (1977) Detection of influential observation in linear regression. Technometrics 19:15–18. doi: 10.1080/00401706.1977.10489493
